# Oral therapy with colonization factor antigen I prevents development of type 1 diabetes in Non-obese Diabetic mice

**DOI:** 10.1038/s41598-020-62881-4

**Published:** 2020-04-09

**Authors:** Andrew S. Nelson, Massimo Maddaloni, Jeffrey R. Abbott, Carol Hoffman, Ali Akgul, Christina Ohland, Raad Z. Gharaibeh, Christian Jobin, Todd M. Brusko, David W. Pascual

**Affiliations:** 1 Department of Infectious Diseases and Immunology, University of Florida, Gainesville, FL United States; 2 Department of Comparative, Diagnostic, and Population Medicine, University of Florida, Gainesville, FL United States; 3 Division of Gastroenterology, Hepatology, and Nutrition, Department of Medicine, University of Florida, Gainesville, FL United States; 4 Department of Pathology, Immunology, & Laboratory Medicine, University of Florida Diabetes Institute, University of Florida, Gainesville, FL United States

**Keywords:** Immunotherapy, Chronic inflammation

## Abstract

Antigen (Ag)-specific tolerization prevents type 1 diabetes (T1D) in non-obese diabetic (NOD) mice but proved less effective in humans. Several auto-Ags are fundamental to disease development, suggesting T1D etiology is heterogeneous and may limit the effectiveness of Ag-specific therapies to distinct disease endotypes. Colonization factor antigen I (CFA/I) fimbriae from *Escherichia coli* can inhibit autoimmune diseases in murine models by inducing bystander tolerance. To test if Ag-independent stimulation of regulatory T cells (Tregs) can prevent T1D onset, groups of NOD mice were orally treated with *Lactococcus lactis* (LL) expressing CFA/I. LL-CFA/I treatment beginning at 6 weeks of age reduced disease incidence by 50% (*p* < 0.05) and increased splenic Tregs producing both IL-10 and IFN-γ 8-fold (*p* < 0.005) compared to LL-vehicle treated controls. To further describe the role of these Tregs in preventing T1D, protective phenotypes were examined at different time-points. LL-CFA/I treatment suppressed splenic TNF-α^+^CD8^+^ T cells 6-fold at 11 weeks (*p* < 0.005) and promoted a distinct microbiome. At 17 weeks, IFN-γ^+^CD4^+^ T cells were suppressed 10-fold (*p* < 0.005), and at 30 weeks, pancreatic Tbet^+^CD4^+^ T cells were suppressed (*p* < 0.05). These results show oral delivery of modified commensal organisms, such as LL-CFA/I, may be harnessed to restrict Th1 cell-mediated immunity and protect against T1D.

## Introduction

Type 1 diabetes (T1D) is a chronic autoimmune disease characterized by host-mediated destruction or loss of functional pancreas β-cells with consequential deficiency in the ability to produce insulin and control blood glucose levels^1^. T1D patients have substantial risk of severe complications including kidney disease, nerve damage, blindness^2^, and cardiovascular disease^3^, collectively resulting in reduced average lifespan^4^. T1D is primarily screened in patients by testing blood glucose levels, which limits diagnosis to a disease stage wherein β-cell function or destruction has already reached a critical level. This has sparked intense study to reveal additional disease biomarkers. Lower overall microbiome diversity and an increasing ratio of Bacteroidetes to Firmicutes over time have been observed in both diabetic humans and mice^5^. In humans, these changes can be detected prior to presentation of islet autoantibodies (AAbs)^6^, signaling the microbiome’s potential as a disease marker^7^.

IAA and/or GADA appear prior to T1D onset in the vast majority of patients, suggesting an ongoing Ag-specific autoimmune attack directed against the pancreatic β-cells^8,9^. T cells specific for insulin and GAD epitopes have been found in both humans^10,11^ and NOD mice^12,13^, one of the most used and best described models of human T1D^14^. This has prompted testing of oral therapies aimed at tolerance induction^15^ taking advantage of gut-associated lymphoreticular tissue (GALT), which specializes in inducing tolerance toward benign foreign Ags, such as food and commensal microbes^16^. Additionally, oral tolerance therapy has an excellent safety profile in human trials for food allergies^17^. Trials with oral insulin showed only a delay in T1D progression in a subset of individuals with high IAA titers, determined by post-hoc analysis^18^, and a larger follow-up trial demonstrated oral insulin to have no significant effect on T1D progression. A more recent study enrolled participants who were relatives of patients with T1D with multiple AAbs present, but prior to diagnosis^19^ and found oral insulin did not delay onset of T1D in most patients. However, treatment was able to delay disease by 31 months in participants with low first-phase insulin release, suggesting that Ag-specific tolerance is effective against certain disease endotypes^19^.

The heterogeneous nature of T1D is a likely reason for the limited efficacy of antigen-specific therapies in humans^20,21^. One candidate to overcome this limitation involves the induction of bystander tolerance through Ag-specific Tregs that directly suppress immune responses toward an irrelevant Ag and indirectly, autoreactive T cells^22^. The studies presented here utilize CFA/I fimbriae to induce bystander tolerance. CFA/I fimbriae are an important virulence factor of enterotoxigenic *Escherichia coli*, though it has been shown to induce anti-inflammatory cytokine responses when expressed by a *Salmonella* vaccine vector (*Salmonella*-CFA/I)^23^ or when the recombinant protein is administered orally^24^. This anti-inflammatory potential has been further studied in the context of experimental models of multiple sclerosis and rheumatoid arthritis. In both the experimental autoimmune encephalomyelitis (EAE) model of multiple sclerosis^25^ and the collagen-induced arthritis (CIA) models^24,26^, oral *Salmonella*-CFA/I protects mice against autoimmune pathology. Such treatment stimulates the induction of CFA/I-specific Tregs, which in a bystander fashion, stimulate auto-Ag-specific Tregs, protecting against autoimmune attack^27^. Notably, the observed Treg phenotypes are unique to the intended disease. In the EAE model, TGF-β-producing Foxp3^+^CD25^+^CD4^+^ Tregs are induced and protective^25^. Protection against CIA is mediated by two distinct subsets of CD39^+^CD4^+^ Tregs: an IL-10-producing Foxp3^+^ population and a TGF-β-producing Foxp3^-^ population^28^. Importantly, CFA/I fimbriae do not act as a broad immunosuppressant; additional studies showed that mice previously treated with *Salmonella*-CFA/I retain the ability to protect against oral, virulent *Salmonella* infection^29^. We hypothesize that CFA/I fimbriae may function more broadly to treat other autoimmune diseases, such as T1D.

Expansion of Tregs, independent of Ag, to effectively prevent and reverse T1D in the NOD mouse, and IL-10 and TGF-β are critical for protection in Treg-mediated therapies^30,31^. These findings suggest that the non-colonizing LL vector^32^, which is generally recognized as safe (GRAS) by the United States Food and Drug Administration (FDA)^33^, expressing CFA/I is an excellent therapeutic candidate for treating T1D. To develop CFA/I fimbriae as an oral, food-based therapy, *Lactococcus lactis* (LL) has been adapted as a delivery vector. LL has long been used for food production. Additionally, compared to *Salmonella*-CFA/I, LL expressing CFA/I (LL-CFA/I) has been shown to elicit the same level of protection and Treg profile in the CIA model of arthritis^34^. Herein, we evaluate the ability of LL-CFA/I to induce Tregs and thereby, protect NOD mice against T1D.

## Results

### Oral Treatment with LL-CFA/I Ameliorates T1D in NOD Mice

**Figure 1:**
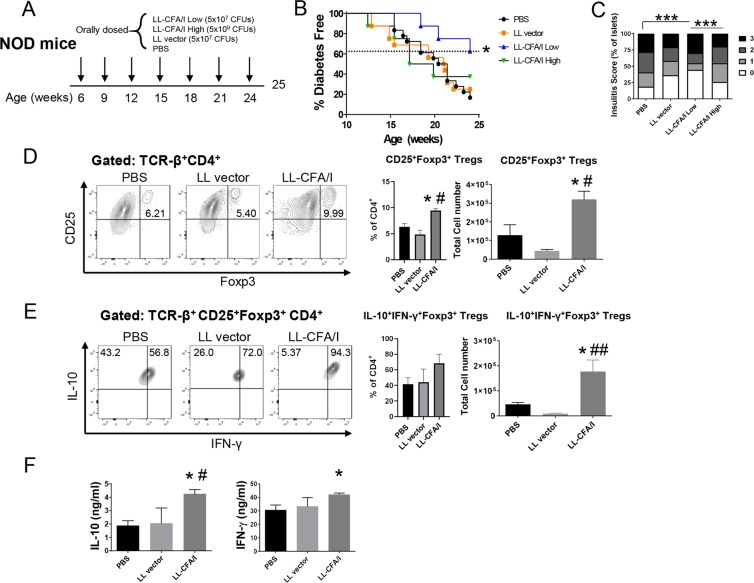
Oral treatment with LL-CFA/I ameliorates T1D in NOD mice. (**A**) Six wk-old female NOD mice were orally dosed with 5 × 10^7^ CFUs of LL CFA/I or LL vector, 5 × 10^9^ CFUs of LL-CFA/I, or PBS (n = 8/group). Additional doses were administered every 3 wks. (**B)** Incidence of T1D in mice given indicated treatments, *p < 0.05 for LL-CFA/I (low dose) vs LL-CFA/I (high dose), LL vector, and PBS groups; and (**C**) frequency of observed insulitis scores of treated mice, ***p < 0.00005 as indicated between PBS and LL-CFA/I (low dose) and between LL-CFA/I low and high doses. (**D**–**F**) At 25 wks, splenic mononuclear cells from surviving mice [PBS (n = 3), LL vector (n = 6), and LL-CFA/I (n = 8)] were purified and cultured in the presence of anti-CD3 and anti-CD28 mAbs for 72 hours. (**D**) Representative plots of CD25^+^Foxp3^+^CD4^+^ T cells (left) and total number of CD25^+^Foxp3^+^CD4^+^ T cells (right). (**E**) Representative plots of IL-10^+^IFN-γ^+^Foxp3^+^CD4^+^ T cells (left) and total number of IL-10^+^IFN-γ^+^Foxp3^+^ Tregs (right). **(F**) Culture supernatants were analyzed for production of IL-10 (left) and IFN-γ (right). Depicted are the means ± SEM; *p < 0.05 for LL-CFA/I vs PBS; ^#^*p* < 0.05, ^##^*p* < 0.005 for LL-CFA/I vs LL vector.

Groups of female NOD mice were orally dosed with PBS, LL carrying the empty pMSP3535 vector (LL vector), or LL-CFA/I. Two doses of LL-CFA/I were tested: a high dose of 5 × 10^9^ and a low dose of 5 × 10^7^ colony forming units (CFUs). Mice were initially dosed at 6 weeks of age and additional doses administered every 3 weeks (Fig. 1A). The low dose regimen significantly reduced T1D incidence in NOD mice: 62.5% of mice given low-dose LL-CFA/I were protected from T1D at 24 weeks of age, as compared to 16.7% and 25% in PBS- and LL vector-treated mice, respectively (*p* < 0.05; Fig. 1B). For mice that received high-dose LL-CFA/I, 37.5% were diabetes-free at the end of the study, which is not significantly different from the control groups (Fig. 1B). Hence, all subsequent studies used 5 × 10^7^ CFUs of LL-CFA/I or LL vector.

At 24 weeks of age, the study was terminated and pancreata collected for histological analysis. Low-dose LL-CFA/I, but not high-dose or LL vector, significantly reduced insulitis levels compared to PBS treatment (*p* < 0.00005, Fig. 1C).

Previous studies utilizing CFA/I based therapies showed a critical role for Tregs in protecting against autoimmunity in animal models^26,35^. Flow cytometric analysis of splenocytes showed ~2.5-fold increase in Foxp3^+^CD25^+^CD4^+^ Tregs compared to the PBS group and>7–fold increase compared to the LL vector group (*p* < 0.05, Fig. 1D). These Tregs were found to produce both IFN-γ and IL-10, with LL-CFA/I inducing a 20-fold increase in IFN-γ^+^IL-10^+^Foxp3^+^CD25^+^CD4^+^ Tregs over the LL vector group (*p* < 0.005, Fig. 1E). Cytokine ELISAs of culture supernatants support the flow cytometry findings with >2-fold increase in IL-10 production (*p* < 0.05) and a significant (*p* < 0.05) increase in IFN-γ production (Fig. 1F). These data show that oral treatment with CFA/I fimbriae protects NOD mice from T1D and induces Tregs of a previously described Tr1-phenotype^36^.

### Optimization of LL-CFA/I Treatment Regimen

**Figure 2:**
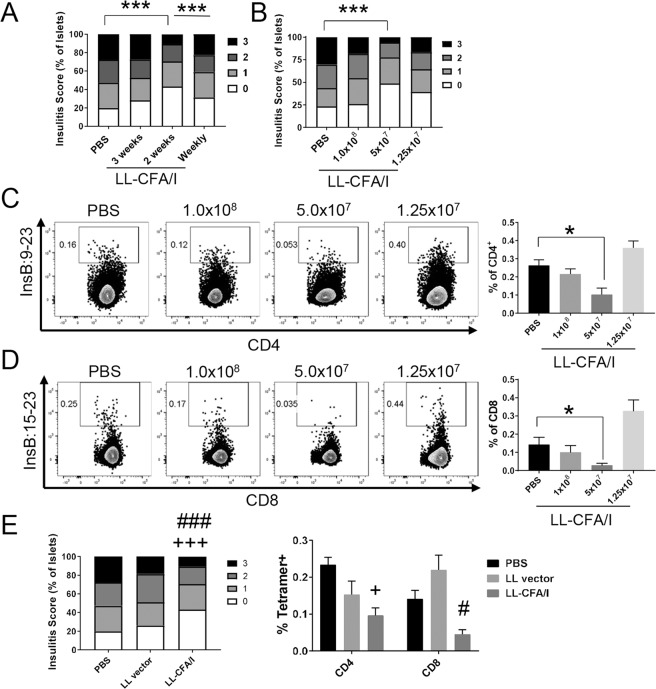
Optimization of therapy with LL-CFA/I. Four wk-old NOD females were dosed with LL-CFA/I or PBS in varying doses or frequencies. At 11 wks of age, 1 wk after the final dose, mice were euthanized and insulitis levels examined. (**A**) Frequencies of observed insulitis scores of mice dosed with 5 × 10^7^ CFUs every 1 (n = 17), 2 (n = 17), or 3 wks (n = 7); ***p < 0.00005 as indicated between PBS and LL-CFA/I treated every 2 wks and between mice treated weekly and every 2 wks with LL-CFA/I. (**B**) Frequencies of observed insulitis scores of mice dosed with 1 × 10^8^ (n = 7), 5 × 10^7^ (n = 17), or 1.25 × 10^7^ (n = 7) CFUs of LL-CFA/I every 2 wks; ***p < 0.00005 as indicated between PBS and 5 × 10^7^ CFUs LL-CFA/I. Insulin-specific (**C**) reduction in diabetogenic CD4^+^ and (**D**) CD8^+^ T cells from females treated with the various doses of LL-CFA/I were observed using tetramers specific to InsB:9-23 and InsB:15-23 (n = 5/group), respectively; ^*^p < 0.05 between PBS and LL-CFA/I (5 × 10^7^ CFUs). (**E**) Frequencies of observed insulitis scores (n = 8/group), left, and summary of insulin-specific CD4^+^ and CD8^+^ T cells from mice dosed with 5 × 10^7^ CFUs of LL-CFA/I, LL vector, or PBS every 2 wks (n = 7/group), right; ^+^*p* < 0.05 and ^+++^*p* < 0.00005 between LL-CFA/I and PBS; ^#^*p* < 0.05 and ^###^*p* < 0.00005 between LL-CFA/I and LL vector^.^ Data are represented as means ± SEM.

To further optimize the dosing regimen, NOD females were orally administered 5 × 10^7^ CFUs of LL-CFA/I or PBS beginning at 4 weeks of age with additional doses given every 3 weeks, every 2 weeks, or weekly until mice reached 10 weeks of age. At 11 weeks of age, pancreata were collected and insulitis levels examined for cross-sectional analysis of therapeutic efficacy. Administering LL-CFA/I every 2 weeks, but not every 3 weeks or weekly significantly (*p* < 0.00005) reduced the average insulitis score as compared to PBS treated mice (Fig. 2A), suggesting that dosing every 2 weeks may provide superior protection.

Therapy was further optimized by testing different doses of LL-CFA/I (1 × 10^8^, 5 × 10^7^, or 1.5 × 10^7^ CFUs or PBS vehicle control) given to female NOD mice every 2 weeks from 4–10 weeks of age. At 11 weeks of age, insulitis was examined. Dosing every 2 weeks with 5 × 10^7^ CFUs, but not with 1 × 10^8^ or 1.5 × 10^7^ CFUs, significantly reduced insulitis scores as compared to the PBS only group (*p* < 0.00005, Fig. 2B). In support of these data, lymphocytes from the pancreatic lymph nodes (PaLNs) were examined for insulin specificity. CD4^+^ T cells were labeled with tetramers specific for insulin B:9–23 (Fig. 2C), and CD8^+^ T cells for insulin B:15–23 (Fig. 2D), or as negative controls, cells were stained with human class II-associated invariant chain peptide 103–107 (CLIP) for CD4^+^ T cells (Supplemental Fig. 1A) or TUM peptide for CD8^+^ T cells (Supplemental Fig. 1B). Flow cytometric analysis of PaLN cells showed a 2-fold reduction in insulin B:9–23-specific CD4^+^ (*p* < 0.05, Fig. 2C) and CD8^+^ T cells (Fig. 2D) in mice treated with 5 × 10^7^ CFUs of LL-CFA/I.

Together, these data show that dosing with 5 × 10^7^ CFUs LL-CFA/I every 2 weeks provides superior protection against autoimmune pathogenesis related to T1D. To confirm these findings, mice were dosed with LL-CFA/I or LL vector beginning at 4 weeks of age with additional doses given every 2 weeks from 4–10 weeks of age. Treatment with LL-CFA/I, but not LL vector, significantly (*p* < 0.00005) reduced insulitis scores by 1.6-fold, and insulin-specific CD8^+^ T cells (*p* < 0.05) by 3.1-fold as compared to the PBS group (Fig. 2E).

### LL-CFA/I Suppresses Tbet-mediated Inflammation at 11 Weeks

**Figure 3:**
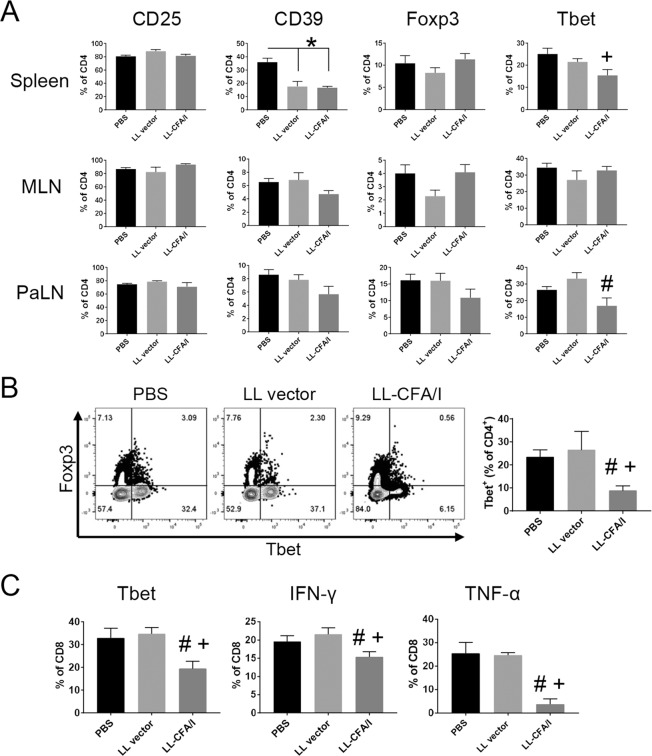
LL-CFA/I suppresses Tbet mediated inflammation at 11 weeks. Four wk-old NOD females were orally dosed with 5 × 10^7^ CFUs of LL-CFA/I, LL vector, or PBS. Additional doses were given every 2 wks. (**A**) At 11 wks of age, lymphocytes from the spleens, MLNs, and PaLNs were stimulated with anti-CD3 and anti-CD28 mAbs for 48 hours. CD4^+^ Tregs from the spleens (n = 12/group), MLNs (n = 8/group), and PaLNs (n = 5/group) were evaluated by flow cytometry for CD25, CD39, Foxp3, and Tbet expression. (**B**) Representative plots of Foxp3 and Tbet expression by pancreatic CD4^+^ T cells (left) and summary of Tbet expression (right) (n = 6/group) are depicted. (**C**) Splenic CD8^+^ T cells from treated mice (n = 12/group) were examined by flow cytometry for Tbet, IFN-γ, and TNF-α expression. Data are represented as means ± SEM. *p < 0.05 for PBS vs LL-CFA/I and LL vector; ^#^*p* < 0.05 for LL-CFA/I vs LL vector; ^+^*p* < 0.05 for LL-CFA/I vs PBS.

A cross-sectional analysis of various lymphoid tissues was performed at 11 weeks to further characterize Tregs induced by LL-CFA/I therapy. Following oral treatment of NOD females with LL-CFA/I every two weeks beginning at 4 weeks of age, lymphocytes from the spleen, mesenteric lymph nodes (MLNs), and PaLNs were stimulated with anti-CD3 and anti-CD28 mAbs, and then analyzed by flow cytometry. Following stimulation, the frequency of CD25^+^CD4^+^ T cells was unchanged, but CD39^+^CD4^+^ T cells was significantly lower in the spleens of animals treated with LL vector or LL-CFA/I compared to the PBS control group, suggesting LL may suppress CD4^+^ T effector (Teff) cell activation in NOD mice (*p* < 0.05; Fig. 3A). The frequency of Foxp3^+^CD4^+^ T cells did not change significantly in any tissue examined as a result of this short-course oral treatment (Fig. 3A). However, LL-CFA/I therapy significantly reduced the frequency of Tbet-expressing CD4^+^ T cells in spleens and PaLNs (*p* < 0.05, Fig. 3A). Tbet^+^CD4^+^ T cells were also reduced in the pancreas (Fig. 3B), but again, no increase in Foxp3^+^CD4^+^ T cell frequency was observed (*p* < 0.05, Fig. 3A).

Previously, *Salmonella*-CFA/I was shown to induce CTLA-4 expression on Tregs in addition to an enhanced cytokine profile^37^. We also assessed the frequency of Foxp3^+^CD4^+^ Tregs expressing co-inhibitory molecules PD-1, CTLA-4 and Tigit. Unlike in the EAE model^27^, LL-CFA/I was found not to induce Tregs expressing these negative regulators in NOD spleens (Supplemental Fig. 2A) or MLNs (Supplemental Fig. 2B). However, the percentages of splenic CD8^+^ T cells expressing Tbet, IFN-γ, or TNF-α were significantly reduced following LL-CFA/I treatment, suggesting the peripheral suppression of activated, proinflammatory CD8^+^ T cells (*p* < 0.05, Fig. 3C). These results show that LL-CFA/I suppresses Th1 cell-mediated inflammation in NOD mice.

Regulation of inflammatory CD4^+^ and CD8^+^ T cells occurs shortly after dosing. To determine if LL-CFA/I influences the innate immune system, expression of cytokines was examined in the intestines and Peyer’s patches shortly after oral dosing of BALB/c mice. Analysis of gene expression shows that treatment with LL-CFA/I upregulates IL-10 (*p* < 0.05) and downregulates inflammatory mediators IL-6, IL-33, and TNFα in the intestines (*p* < 0.005; Supplemental Fig. 3A). Interestingly, this pattern is not evident in the Peyer’s Patches, suggesting that LL-CFA/I influences cells in the gut epithelium or the gut microbiota itself.

### LL-CFA/I Alters NOD Gut Microbiota

**Figure 4:**
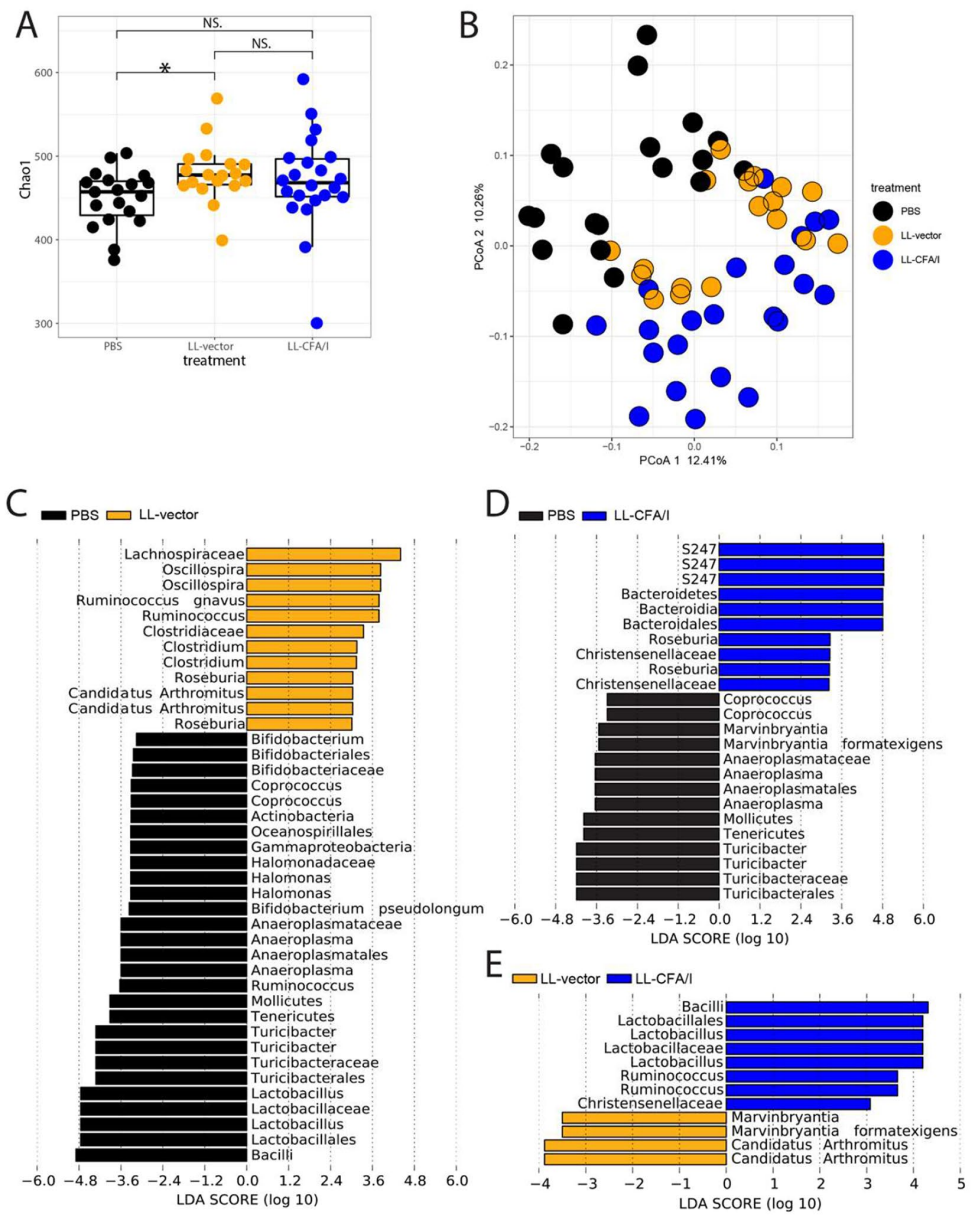
Treatment with LL-CFA/I modulates the gut microbiota. Alpha diversity calculations (**A**) and beta diversity principal coordinates analysis (PCoA; **B**) of fecal bacteria 16 S rDNA from 11 week-old NOD females treated with PBS, LL-vector, or LL-CFA/I, vs LL-vector Axis 1 P = 0.03, PBS vs LL-CFA/I Axis 1 P = 0.02, LL-vector vs LL-CFA/I Axis 2 P = 0.008. Linear discriminant analysis (LDA) plots show taxa that are differentially abundant between mice treated with PBS and LL-vector (**C**), PBS and LL-CFA/I (**D)**, or LL-vector and LL-CFA/I (**E**). *p < 0.05; NS, not significant.

To investigate whether LL-CFA/I influences the intestinal microbiota, female NOD mice were administered 5 × 10^7^ CFUs of LL-vector, LL-CFA/I, or PBS every two weeks beginning at 4 weeks of age, and fecal samples were collected at 4 weeks of age (prior to treatment) and at 11 weeks of age (one week after the fourth oral treatment) for 16 S rDNA sequencing of mouse fecal bacteria. Before treatments began, we found no significant differences in the microbial communities across the groups in terms of alpha- or beta-diversity (Supplemental Fig. 4B,C). At 11 weeks, alpha diversity was significantly increased by LL vector treatment compared to PBS control, but not by the LL-CFA/I (Fig. 4A). Of note, principal coordinates analysis (PCoA) showed distinct, statistically significant clustering across the treatment groups (Axis 1 P = 0.02; Fig. 4B) as well as for pairwise comparison between groups (Supplemental Fig. 4A). This indicates that the bacterial gavage itself alters the microbiome, which is further shifted by the presence of the CFA/I plasmid. Linear discriminant analysis (LDA) revealed differential abundance of many bacterial taxa among the groups (Fig. 4C–E), most notably an enrichment of *Lactobacilli* and *Ruminococcus*, which have been described as beneficial to human health^38,39^, in mice treated with LL-CFA/I compared to those given LL vector (Fig. 4E). We examined microbial communities before treatments began (from 4-week-old mice) and found no significant different across the groups in terms of alpha- or beta-diversity (Supplemental Fig. 4B,C). This suggests that the change in the microbial community is caused by the treatments and not due to differences in the initial gut microbiota.

### LL-CFA/I-Induced Tregs Maintain Suppressive Phenotype at 17 Weeks

**Figure 5:**
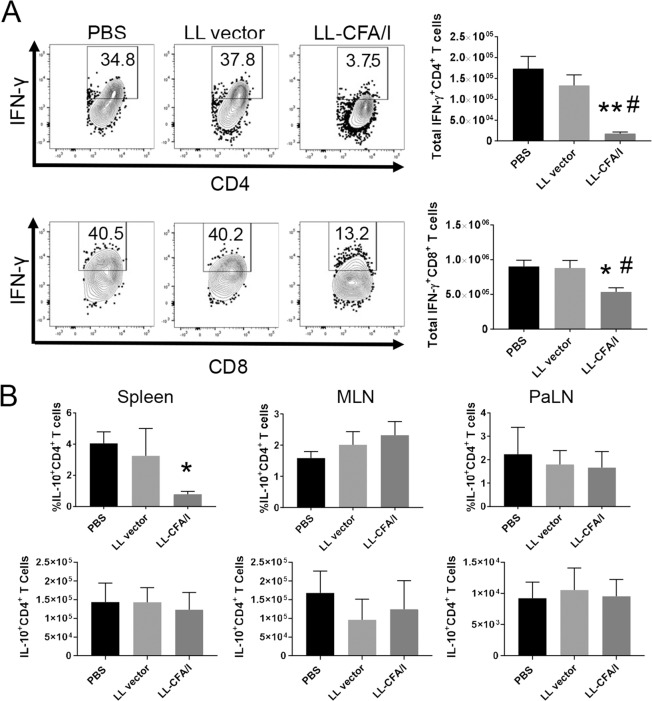
Treatment with LL-CFA/I suppresses IFN-γ-mediated inflammation at 17 weeks. Four week-old NOD females (n = 10/group) were orally dosed with 5 × 10^7^ CFUs of LL-CFA/I, LL vector, or PBS. Additional doses were given every 2 wks. At 17 weeks, one week after their final dose, density gradient-purified lymphocytes from spleens, MLNs and PaLNs were stimulated with anti-CD3 and anti-CD28 mAbs. (**A)** Representative plots of IFN-γ expression by (**A**) splenic CD4^+^ and (**B**) splenic CD8^+^ T cells (left), and total numbers of IFN-γ-producing (**A)** CD4^+^ and (**B**) CD8^+^ T cells (right) are shown. (**C)** The percentage (top row) and total (bottom row) of IL-10-producing CD4^+^ T cells in spleen, MLNs, and PaLNs are depicted. Data are represented as means ± SEM; *p < 0.05 and ^**^p < 0.005 for LL-CFA/I vs PBS; ^#^*p* < 0.05 for LL-CFA/I vs LL vector.

To elucidate how the protective response generated by LL-CFA/I treatment evolves during disease pathogenesis, NOD mice were treated with PBS, LL vector, or LL-CFA/I (5 × 10^7^ CFUs every two weeks) from 4–16 weeks of age, and Teff and Treg phenotypes were examined in spleens and PaLNs at 17 weeks of age. Notably, treatment with LL-CFA/I reduced splenic IFN-γ^+^CD4^+^ T cells by 10-fold (*p* < 0.005; Fig. 5A upper panel). Splenic IFN-γ^+^ CD8^+^ T cells were also significantly reduced (*p* < 0.05; Fig. 5A lower panel). These data show that the Th1 cell-targeted suppression following CFA/I treatment is stable out to 17 weeks.

**Figure 6:**
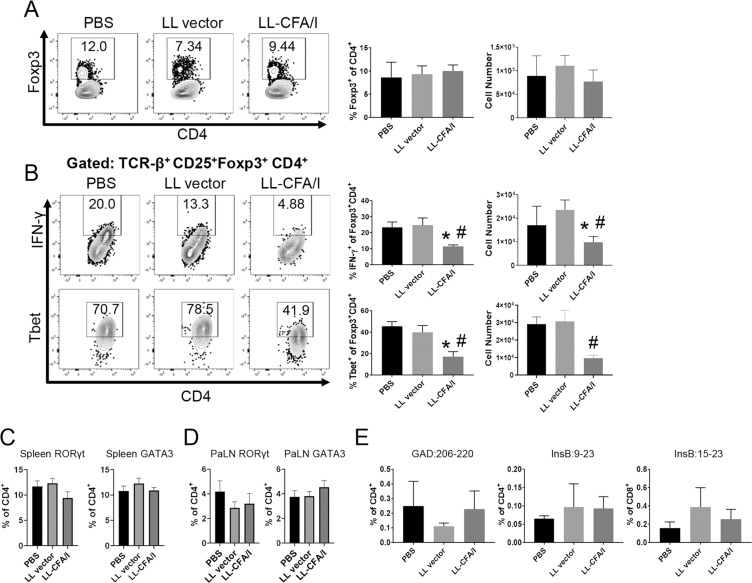
LL-CFA/I maintains suppressive Tregs in the PaLNs at 17 weeks. Four wk-old NOD females were orally dosed with 5 × 10^7^ CFUs of LL-CFA/I, LL vector, or PBS per the treatment regimen described in Fig. 5. (**A**,**B**) At 17 wks, one wk after their final dose, purified PaLN lymphocytes were stimulated with anti-CD3 and anti-CD28 mAbs to assess by flow cytometry (**A**) Foxp3^+^CD4^+^ Tregs and co-expression of (B) IFN-γ and Tbet. Expression of RoRγt and GATA3 by splenic (**C**,**D**) PaLN CD4^+^ T cells was also measured. (**E**) Staining was performed on unstimulated diabetogenic PaLN T cells to detect tetramer-specific GAD:206-220 and InsB:9-23 CD4^+^ T cells, and tetramer-specific InsB:15-23 CD8^+^ T cells. Data are represented as means ± SEM; *p < 0.05 for LL-CFA/I vs PBS; ^#^*p* < 0.05 for LL-CFA/I vs LL vector.

Similar to the 11-week data (Fig. 3A, bottom), at 17 weeks of age, PaLN lymphocytes stimulated with anti-CD3 and anti-CD28 mAbs displayed no significant differences in the frequency of Foxp3^+^CD4^+^ T cells, suggesting that Treg induction in response to LL-CFA/I treatment (Fig. 1E) may not be evident as early as 17 weeks (Fig. 6A). Moreover, there was no observed change in splenic IFN-γ^+^IL-10^+^ Treg frequency at 17 weeks of age (Supplemental Fig. 5A). Notably, IFN-γ^+^Foxp3^+^ and Tbet^+^Foxp3^+^ Tregs were more frequent in PBS or LL vector treated mice, suggesting their suppressive function may be compromised and that they had taken on a proinflammatory phenotype (Fig. 6B). However, cells expressing these inflammatory mediators, IFN-γ or Tbet, were significantly reduced in the LL-CFA/I group (Fig. 6B). Since IL-10 was not upregulated in the PaLNs at this time-point (Fig. 5B), Th2 and Th17 cell phenotypes were examined. The percentages of splenic and PaLN CD4^+^ T cells expressing the Th17 and Th2 cell transcription factors, RoRγt and GATA3, were not altered by LL-CFA/I treatment (Fig. 6C,D), suggesting that suppression of IFN-γ-producing cells by LL-CFA/I treatment was not associated with compensatory increases in Th2 or Th17 cells.

Tetramer staining for insulin and GAD-reactive CD4^+^ and CD8^+^ T cells in the PaLNs showed no significant differences across the three treatment groups (Fig. 6E). This may be due to the Ag-independent manner in which LL-CFA/I protects the mice. However, since this is a later disease time-point than what was observed in previous experiments it is possible that epitope spreading is playing a role. Others have demonstrated epitope spreading does occur for T1D in both mice and humans, and this may dilute the detection of insulin- or GAD-specific T cells^40,41^.

### Optimized LL-CFA/I therapy is protective long-term

**Figure 7:**
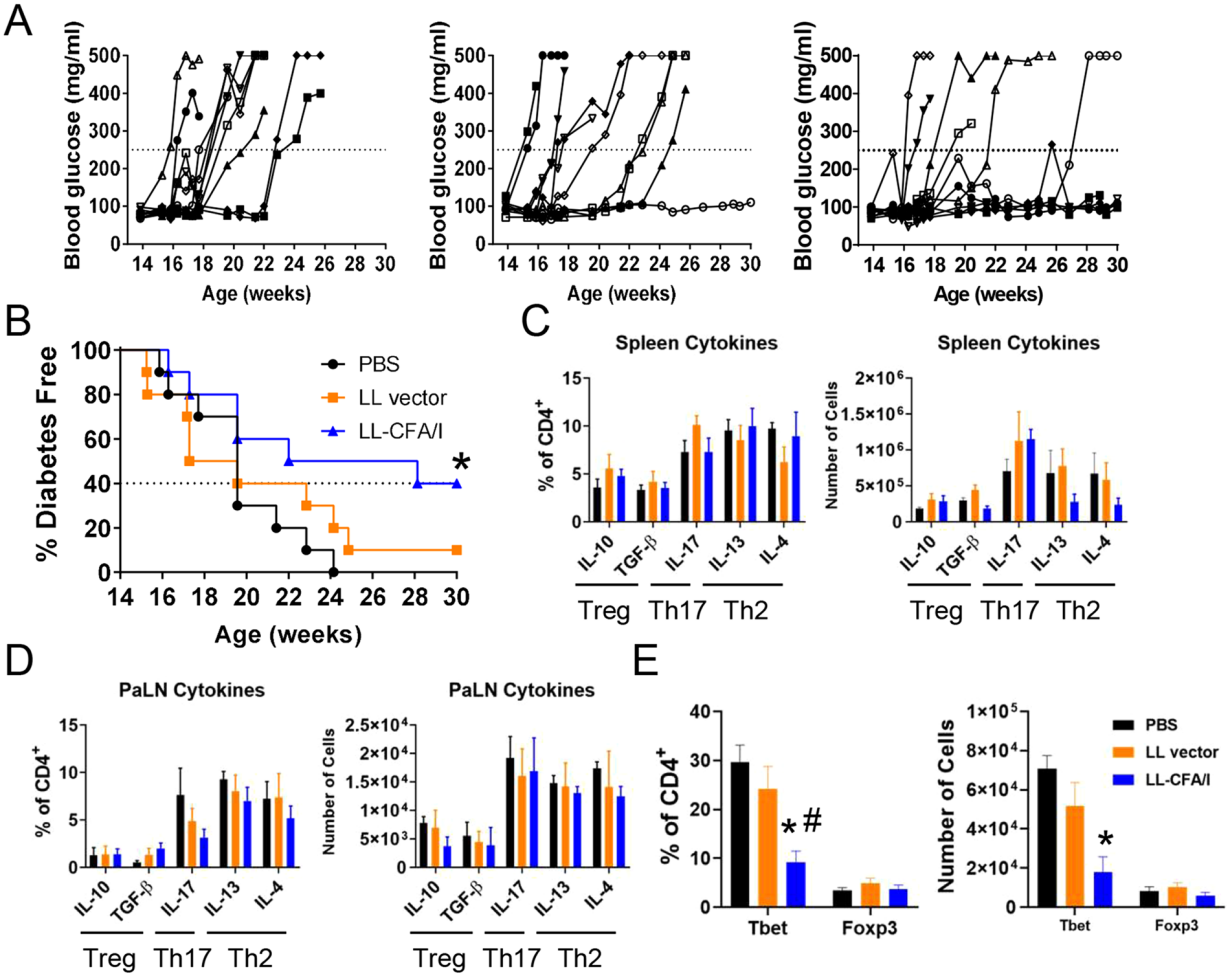
Optimized LL-CFA/I therapy is protective at 30 wks. Four wk-old NOD females were orally dosed with 5 × 10^7^ CFUs of LL-CFA/I, LL vector, or PBS. Additional doses were given every 2 wks. (**A)** Blood glucose levels of individual mice treated with LL-CFA/I, LL vector, or PBS (n = 10/group) are depicted. Values above the dotted lines are considered hyperglycemic. (**B**) Summary of disease incidence of mice given the various treatments. Dotted line represents incidence of mice treated with LL-CFA/I. At 30 wks or when hyperglycemic mice were euthanized, (**C)** splenic and (**D**) PaLN lymphocytes were stimulated with anti-CD3 and anti-CD28 mAbs to assess extent of IL-10, TGF-β, IL-17, IL-13, and IL-4 production. Pancreatic lymphocytes were purified and analyzed for expression of (**E**) Foxp3 and Tbet expression; PBS (n = 5), LL vector (n = 5), and LL-CFA/I (n = 7). *p < 0.05 for LL-CFA/I vs PBS; ^#^*p* < 0.05 for LL-CFA/I vs LL vector.

To test for the durability of LL-CFA/I’s protective efficacy, 4 week-old NOD females were orally dosed with 5 × 10^7^ CFUs of LL-CFA/I or LL vector in PBS or vehicle alone every 2 weeks until diabetes onset or 30 weeks of age, at which time, spleens and PaLNs were harvested for flow cytometric analysis. Treatment with LL-CFA/I significantly reduced the incidence of T1D, with 40% of mice remaining normo-glycemic (*p* < 0.05; Fig. 7A,B). Lymphocytes from spleens and PaLNs were stimulated with anti-CD3 and anti-CD28 mAbs, and cytokine profiles were examined by flow cytometry. There was no significant differences in the frequency or number of cells expressing IL-10, TGF-β, IL-17, IL-13, or IL-4 suggesting that Treg, Th2, and Th17 cell cytokines were not altered by LL-CFA/I in the context of T1D (Fig. 7C,D). Interestingly, within islet-resident CD4^+^ T cells, Foxp3^+^CD4^+^ T cells were not found to differ amongst the three treatment groups (Fig. 7E), but Tbet^+^CD4^+^ T cells were reduced in mice treated with LL-CFA/I (Fig. 7E). These results suggest LL-CFA/I maintains suppression of Th1 cell-based inflammation late into disease.

## Discussion

A heterogeneous pathogenesis poses many challenges for therapies that target specific Ags for tolerization, a single cell type or cytokine. Inducing bystander tolerance may offer a safe and effective alternative without the limitations associated with broad immunosuppression (off-target effects) or personalized therapy (feasibility).

Oral treatment with CFA/I fimbriae was shown to prevent autoimmune disease in induced murine models of multiple sclerosis and arthritis^26,35^, and protection was ultimately shown to be dependent upon Tregs with Ag-specificity toward disease relevant Ags. Notably, the phenotypes of these Tregs varied between EAE and CIA models. In EAE, TGF-β-producing Foxp3^+^CD25^+^CD4^+^ Tregs were shown to play a definitive protective role^25^. Conversely, protection against CIA was mediated via two distinct populations of CD39^+^ Tregs, one being an IL-10-producing Foxp3^+^ population, and the other a TGF-β-producing Foxp3^-^ population^28^. These findings suggest that CFA/I-induced bystander suppression can stimulate different Treg subsets uniquely equipped to suppress inflammation in the context of the specific inflammatory diseases. Importantly, treatment with *Salmonella*-CFA/I did not impair the ability to resist a wild-type *Salmonella* challenge, suggesting CFA/I fimbriae offer a uniquely powerful means to treat autoimmune diseases without immunocompromising the host^29^.

As T cells are the dominant component of insulitis in NOD mice by 11 weeks of age^42^, cross-sectional studies at the 11 week time-point were performed to examine the potential mechanisms of LL-CFA/I therapy. Indeed, treatment with LL-CFA/I significantly reduced insulitis and insulin-reactive CD4^+^ and CD8^+^ T cells in the PaLNs at 11 weeks. Additionally, Tbet-expressing CD4^+^ T cells were suppressed in the PaLNs and pancreas with further suppression of inflammatory CD8^+^ T cells observed in the spleen. Our data suggest that systemic suppression of Tbet and IFN-γ-producing CD4^+^ and CD8^+^ T cells may be a sign of protection. Notably, these findings were different than what was observed at 24 weeks of age, when splenic Tr1 cells were observed. Though, Tregs are canonically identified by expression of Foxp3 and CD25; however, previous studies with CFA/I fimbriae induce canonical Tregs to treat EAE and CD39^+^ Tregs to treat CIA^25,28,43^. The data in this report fit with previously described findings suggests that LL-CFA/I induces Tregs with phenotype and cytokine profile unique to the context of auto-inflammation. Though Treg phenotypes varied between early and late time-points, insulitis scores were consistently reduced. Protection against insulitis may be in part explained by Th1 cell-targeted suppression in the PaLNs observed at 11, 17, and 30 weeks of age, suggesting that treatment with LL-CFA/I specifically suppresses auto-reactive Teffs’ entry into the pancreas. Alternatively, it may stimulate a regulatory environment not conducive for Teff expansion.

T1D is considered to progress over stages defined by the presence of AAbs (Stage 1) followed by the development of dysglycemia (Stage 2) and then, clinical diagnosis (Stage 3)^44^. In our colony, 50% of female NOD mice become diabetic between 16 and 20 weeks of age. Hence, T cell phenotypes were examined at 17 weeks to determine the durability of immunoregulation in response to LL-CFA/I therapy prior to Stage 3 of disease. In agreement with our 11-week data, reduced IFN-γ^+^ T cells in the spleens of LL-CFA/I treated mice persisted to 17 weeks. Additionally, in the PaLNs, Foxp3^+^ Tregs from PBS or LL vector-treated mice expressed Tbet and IFN-γ after anti-CD3/anti-CD28 mAb stimulation, but these populations were reduced in the LL-CFA/I treated group. These data support the hypothesis that Tregs in T1D become ex-Tregs during disease progression^45^ and suggest that LL-CFA/I treatment may serve to sustain Treg-mediated immunoregulation. Long-term LL-CFA/I mediated protection against T1D in NOD mice was similarly associated with suppression of Tbet^+^CD4^+^ Teff cells in the pancreas with no change in the quantity of Foxp3^+^CD4^+^ Tregs at 30 weeks of age. Previous reports have shown, that T1D is not associated with a decline in the number of Foxp3^+^ Tregs, but rather have suggested their function to be impaired^46,47^. It is possible that LL-CFA/I affects the function of Tregs, but not the numbers. This is reflected in data from the gut-associated MLNs, where the number of Foxp3^+^ Tregs was not affected at 11 weeks. This finding was unexpected since the LL-CFA/I was administered orally, where its initial impact should be in the GALT.

Treatment with LL-CFA/I did impact the gut microbiota as distinct gut microbial communities were observed for NOD mice treated with LL-CFA/I, LL vector, or PBS. LL vector treatment significantly altered the diversity of the microbiota and bacteria representation with the community; however, these changes in the microbiota were not associated with reductions in insulitis at 11 weeks, nor T1D incidence at 30 weeks. Notably, *Lactobacilli* were over-represented in mice treated with LL-CFA/I, which has been associated with lower risk of T1D development in rodents and humans^6,48^. Additionally, LL-CFA/I neither increased alpha diversity nor affected the Bacteroidetes to Firmicutes ratio, which are associated with lower risk of T1D in mice^5^.

The gut microbiota plays an important role in shaping the susceptibility to T1D, although the exact mechanisms remain unknown^49^. NOD mice have reduced mucous-producing goblet cells, suggesting a potential mechanism as bacteria or food antigens may translocate to the PaLNs of mice^50^. Our data show LL-CFA/I promotes a regulatory environment within the small intestine, thus, the effect of the microbiota or LL-CFA/I may be initially mediated through interactions with the innate cells within the GALT. Further studies with LL-CFA/I will focus on whether the protective phenotype is mediated through the altered microbiota or through innate cells, such as dendritic cells in the GALT, which may control Treg and Teff cell differentiation during T1D development.

In summary, the data presented here show that CFA/I fimbriae provides robust protection against chronic autoimmune diseases such as T1D. Likewise, this report provides evidence of the benefits of utilizing recombinant *L*. *lactis* as a vector to deliver therapeutics. Previous studies by other groups show that dosing with recombinant *Lactococcus* can prevent or reverse T1D^51,52^, however, these required daily dosing and a pre-treatment with anti-CD3 mAb to deplete T cells. In contrast, dosing with LL-CFA/I only every 2 weeks was enough to reduce disease incidence by half or more. Prevention and reversal of T1D is complicated due to its multiple endotypes and heterogeneous pathogenesis. The data presented here show that LL-CFA/I suppresses Th1 cell-mediated inflammation to protect NOD mice from the heterogeneous, evolving pathogenesis of T1D.

## Methods

### Mice

Female NOD/ShiLtJ mice (3–6 weeks of age) were purchased from The Jackson Laboratory, and were monitored for T1D by tail bleed twice a week. T1D was diagnosed after 2 consecutive blood glucose readings above 250 mg/dL, measured by glucometer (Abbott). Mice were housed under SPF conditions with food and water available *ad libitum*. Mice were allowed to acclimate to the facility for at least 5 days prior to handling. BALB/c mice (Charles River Laboratory, Frederick, MD) were 6–8 weeks of age. All mice ice maintained under specific pathogen-free conditions with food and water provided *ad libitum*. All animal experiments conducted in these described studies are in strict accordance with the recommendations in the Guide for the Care and Use of Laboratory Animals of the National Institutes of Health. All animal procedures were approved by the University of Florida Institutional Animal Care and Use Committee (IACUC).

### Grading Insulitis

Freshly isolated pancreata were formalin-fixed, paraffin-embedded, deparaffinized, rehydrated, and cut in 5 µm sections for staining with H&E. Two sections were cut from each pancreata, 150 µm apart, to provide two distinct views inside each pancreas and to ensure enough islets were present for analysis. Prepared slides were analyzed under a Nikon Eclipse E200 microscope (Nikon, Minato, Tokyo, Japan). At least 20 islets from each pancreas were analyzed. Observed islets were given a score ranging from 0 to 3 based on degree of cellular infiltration as previously described^53^: 0, corresponds to no infiltration; 1, corresponds to peri-insulitis or infiltration limited to the boundary of the islet; 2, corresponds to less than 50% of the islet infiltrated; and 3, corresponds to greater than 50% of the islet infiltrated.

### Preparation of LL-CFA/I and Mouse Dosing

*L*. *lactis* IL1403 was engineered to carry the pMSP3535H3 vector in its empty state or with a synthetic operon for CFA/I fimbriae as described previously^34^. Briefly, starter cultures were grown overnight, expanded, and later induced with 0.5 µg/mL nisin (Sigma-Aldrich). Four hours after induction, NOD and BALB/c mice were first given an oral gavage of 10% sodium bicarbonate (Sigma-Aldrich) in sterile water to neutralize stomach acid. After 10 minutes, mice were orally gavaged with the specified dose of LL-CFA/I or LL vector in PBS, or sterile PBS alone. BALB/c mice were dosed with 2 × 10^9^ CFUs of LL vector or LL-CFA/I. Jejunum (without Peyer’s patches) and Peyer’s patches from individual mice were collected for mRNA analysis 1.5 hrs after LL dosing. Washed tissue samples were immediately placed into RNAlater Stabilization Solution (Sigma-Aldrich) and stored in −20 °C until RNA extraction.

### Cell Culture

Mixed and purified NOD T cell cultures were used to observe changing T cell phenotypes. Lymphocytes were cultured in a complete media (CM); RPMI 1640 containing 10% fetal bovine serum (Atlanta Biologicals, Oakwood, Georgia), 50 µM 2-mercaptoethanol (Sigma-Aldrich), plus supplements (Invitrogen), 2 mM L-glutamine, 100 U/mL penicillin, 100 µg/mL streptomycin, 1 mM sodium pyruvate, and 0.1 mM nonessential amino acids. Cells were cultured at 200,000 cells/well in 96-well, round-bottomed tissue culture plates (MilliporeSigma) coated with 5 µg/mL anti-CD3 mAb (clone 17A2; Invitrogen, Carlsbad, CA, USA). Anti-CD28 mAb (clone 37.51; Invitrogen, Carlsbad, CA, USA) was then added to a final concentration of 2.5 µg/mL and samples incubated for 48 hours at 37 °C. Stimulated cells intended for FACS analysis were treated with brefeldin A (Biovision, San Francisco, CA, USA), at a working concentration of 5 µg/mL, for the last 4 hours of culture. Supernatants of cell cultures meant for cytokine analysis by ELISA were collected by centrifugation and stored at −20 °C until analysis.

### Flow Cytometry

Splenic and LN cells were stained for viability using a LIVE/DEAD Fixable Blue Dead Cell Stain Kit, for UV excitation (ThermoFisher). Cells were then washed with DPBS (Gibco) plus 10% FBS (Atlanta Biologicals**)** – and labeled with mAbs specific for TCR-β, CD4, CD25, CD44, Tigit, Tim-3, TGF-β (BioLegend), CD8α, CD39, PD-1, CXCR3 (eBioscience), CD62L, CD103, and CTLA-4 (Becton Dickinson [BD], Franklin Lakes, NJ) (Supplemental Table 1). Cells were then fixed and permeabilized using the True-Nuclear Transcription Factor Buffer Set (BioLegend, San Diego, CA) and labeled with mAbs specific for IFN-γ, IL-17, IL-4, IL-13 (BD), IL-10, Foxp3, Tbet, Gata-3 (eBioscience), TNF-α and RORγt (BioLegend) (Supplemental Table 1). Fluorescence was acquired on a BD Fortessa flow cytometer with BD FACSDiva software. All samples were analyzed using FlowJo software (BD).

### Staining with Tetramers

MHC class II tetramers specific for InsB:9–23, GAD:206–220, and human CLIP along with MHC class I tetramers specific to InsB:15-23 and TUM peptide (KYQAVTTTL) were provided by the NIH Tetramer Core Facility (Emory University, Atlanta, GA, USA). Single cell suspensions of cells from lymphoid, pancreatic tissues, or whole blood were prepared as described above. One million cells were added to FACS tubes for preparation of compensation controls. Cells were washed and supernatants were discarded before being resuspended in 200 µL of DPBS (Gibco) plus 10% FBS (Atlanta Biologicals). To block nonspecific staining via Fc receptors, 1.25 µg of anti-mouse CD16/32 (ThermoFisher) was added to each tube. Samples were incubated for 10 minutes at RT, protected from light. Without washing, 0.63 µg of a given tetramer (NIH Tetramer Core Facility) was added to each tube. Samples were incubated at RT for 45 minutes, protected from light. Without washing, cells were stained for surface antigens as described above. Samples were then washed and analyzed on a BD Fortessa flow cytometer without fixation.

### Cytokine ELISA

At termination of the 11 or 24 wk studies, spleens and MLNs were aseptically removed from treated mice. Lymphocytes were cultured and stimulated as described above. The supernatants were collected by centrifugation and stored at −80 °C. Cytokine capture ELISAs were employed to quantify duplicate sets of samples for the levels of IFN-γ, IL-10, TGF-β, and IL-17 produced by lymphocytes. The methods used are similar to those previously described^23,30^. Briefly, wells were coated with purified anti-mouse mAbs: anti-IFN-γ (clone R4-6A2, 10 µg/mL; ThermoFisher), anti-IL-10 (clone JES5-2A5, 2 µg/mL; eBioscience), anti-TGF-β (clone A75-22 µg/mL; eBioscience), and anti-IL-17 (clone TC11-18H10, 2 µg/mL; BD). For detection, biotinylated anti-mouse IFN-γ (clone XMG1.2, 0.5 µg/mL; BD), IL-10 (clone SCX-1, 1.5 µg/mL; BD), TGF-β (clone A75-3, 5 µg/mL; BD), and IL-17 (clone TC11-8H4, 1.5 µg/mL; BD) were used. The color reaction was developed using a horseradish peroxidase (HRP) conjugated goat anti-biotin Ab (Vector Laboratories, Burlingame, CA, USA) and ABTS peroxidase substrate (Moss, Inc., Pasadena, ME, USA). Cytokine concentrations were extrapolated from standard curves generated by recombinant murine cytokines IFN-γ (Peprotech, Rocky Hill, NJ, USA), IL-10, TGF-β, and IL-17 (R&D Systems, Minneapolis, MN, USA).

### Isolating Lymphocytes from the Islets of Langerhans

Lymphocytes from the islets of Langerhans were purified as previously described^54^. Briefly, the pancreas was perfused with collagenase type IV (MilliporeSigma) through the common bile duct. Pancreata were mechanically dissociated and islets were handpicked under a dissecting microscope (Leica). Islets were cultured overnight in complete media and lymphocytes collected the following day to analyze by flow cytometry.

### Reverse Transcription and Real-time Quantitative Polymerase Chain Reaction (RT-qPCR)

Total RNA was extracted using a kit (TRIzol® Plus RNA Purification Kit, Life Technologies), and RNeasy® (QIAGEN) mini kit accordance with the manufacturer’s instructions. Quality and quantity of RNA were determined by measuring the absorbance at 260 and 280 nm using NanoDrop ND-1000 UV-Vis Spectrophotometer (ThermoFisher). Contaminating DNA was eliminated by DNase I treatment with RNase-Free DNase Set (Qiagen). First-strand cDNA was produced by using Maxima First Strand cDNA Synthesis Kit for RT-qPCR (ThermoFisher). For gene-expression analysis, quantitative real-time PCR (qRT-PCR) was performed using a 20 µl PCR reaction containing 10 µl SYBR Green 2X mix, 0.2 µM each of forward and reverse primers, and 1 µl of 10 × diluted cDNA (Supplemental Table 2). The PCR was set to initial denaturation at 95 °C for 3 min, 42 cycles of denaturation at 95 °C for 15 s, annealing at 62 °C for 30 s, and extension at 72 °C for 30 s, and a final extension at 72 °C for 3 min. At the end of the PCR, a melting curve program from 60 °C to 95 °C with 0.5 °C increase every 15 s were run. The assessment was done by PCR to determine the suitability of primers and experimental instructions. All samples were tested in triplicate. Differences in the threshold cycle (ΔCt) number were determined between the target genes and the housekeeping genes. The relative induction of mRNA expression was determined after normalization using Beta actin and GAPDH as the reference genes, and the results are shown as relative values of mRNA expression versus that of the control, which was given a value of 1. Primers were designed from nucleotide sequences identified using NCBI BLAST (http://blast.ncbi.nlm.nih.gov/Blast.cgi) to confirm the specificity of the primer design. The primer characteristics of selected genes are listed in Supplemental Table 2.

### rRNA Sequencing

Freshly ejected fecal samples from individual NOD females were collected at 4 weeks (pre-treatment) and 11 weeks of age (post-treatment) and snap frozen. DNA was extracted using phenol/chloroform separation followed by DNeasy Blood & Tissue Kit (Qiagen). Library preparation was performed using primers targeting the V1-V3 hypervariable region of the 16 S rRNA gene (27 F: 5′-AGAGTTTGATCCTGGCTCAG-3′ and 534 R: 5′-ATTACCGCGGCTGCTGG-3′). Primers contained universal Illumina paired-end adaptor sequences and unique 4-6 nucleotide barcodes for multiplex sequencing. PCR products were visualized on an agarose gel, purified with the Agencourt AMPure XP kit (Beckman Coulter), quantified with the KAPA Library Quantification Kit (KAPA Biosystems), and equimolar amounts of each sample pooled for sequencing with Illumina MiSeq (paired-end, read length= 300 bases).

### Analysis of 16S rDNA Sequences

Sequencing reads for the 92 samples were trimmed and filtered at Q20 using the Quantitative Insights into Microbial Ecology (QIIME)^55^ version 1.9.1. The resulting set of reads was fed to QIIME’s pick_open_reference_otus.py to pick OTUs at 97% similarity level using the Greengenes 97% reference dataset (release 13_8). Chimeric sequences were detected and removed using QIIME. OTUs that had ≤ 0.005% of the total number of sequences were excluded according to Bokulich and colleagues^56^. One sample didn’t produce enough reads (<50 reads) and was removed from subsequent steps. The final dataset has an average of 53,239 reads per sample (min= 19,079 reads; max= 90,275 reads). Taxonomic assignment was done using the RDP (ribosomal database project) classifier^57^ through QIIME with confidence set to 50%.

For beta diversity analysis, we generated Principal Coordinate Analysis (PCoA) using the phyloseq R package^58^ from Bray-Curtis dissimilarity matrix using the normalized and log_10_ transformed counts^59^ according to the following formula:

$$lo{g}_{10}\left(\frac{RC}{n}\,{\rm{x}}\,\frac{{\sum }^{}x}{N}+1\right)$$

where *RC* is the read count for a particular OTU in a particular sample, *n* is the total number of reads in that sample, the sum of *x* is the total number of reads in all samples and *N* is the total number of samples.

Difference in the microbial community structure was tested as described previously^59,60^. Briefly, differences were detected using the lme function in the R nlme package, with the REML method to fit a generalized mixed linear model of the following form: axis ~ treatment + 1|cage + ɛ, where axis indicates the PCoA axis, treatment indicates the treatment, and 1|cage indicates that we used the cage as a random effect. Then ANOVA analysis on the above model was performed to generate P values for the treatment. The results from lme were confirmed using PERMANOVA through the vegan R package command adonis (version 2.5) with 1000 permutations.

Alpha diversity (Chao1 diversity index) was calculated using the phyloseq R package from the rarefied counts and differences were tested using the above described lme model using diversity index instead of PCoA axis.

Linear discriminant analysis effect size (LEfSe)^61^ was used to identify biomarkers associated with each treatment and only OTUs with a P-value <0.01 and LDA threshold value> 3 were considered significant.

P values were adjusted for multiple hypothesis testing using the method of Benjamini and Hochberg^62^ when multiple tests were performed.

16S sequencing reads have been deposited in the National Center for Biotechnology Information (NCBI) Sequence Read Archive (SRA) under accession number PRJNA557419.

### Statistics

All presented data are the mean ± standard error of the mean (SEM). Statistical significance was tested using GraphPad Prism 8 (Prism). One-way ANOVA with Tukey’s multiple comparisons test were used to compare FACS data, cell counts, and cytokine production. Chi-squared test for trend with Bonferroni correction was performed to determine differences in insulitis scores. Log-rank (Mantel-Cox) tests were used to compare incidence of T1D. All results were discerned to the 95% confidence interval.

Relative expression values were calculated by the threshold cycle (Ct) changes in sample and control using the ΔΔCt method^63^. All expression values were normalized against average of Beta actin and GAPDH expression. Relative expression was determined by the comparative Ct method of relative quantification (RQ), which was calculated with the formula 2^−ΔΔCt^. ΔΔCt was calculated by ΔΔCt = ΔCt (vaccinated) − ΔCt (not vaccinated PBS), where ΔCt is the normalized signal level in a sample (ΔCt = Ct of target gene − Ct of reference gene). Student’s T test was used to compare gene expression among different conditions with GraphPad Prism 8. P value cutoff for statistical significance was < 0.05. To increase statistical reliability, three technical replicates were run for each sample, and the mean Ct considered for each gene.

## Supplementary information

Supplementary Tables 1-2 & Supplementary Figures 1-5.
