# Supplementary material for: Oral therapy with colonization factor antigen I prevents development of type 1 diabetes in Non-obese Diabetic mice
Source: Sci Rep. 2020 Apr 9;10:6156. doi: 10.1038/s41598-020-62881-4 (PMC7145799; doi:10.1038/s41598-020-62881-4)
Supplement: Supplementary file 1 — Supplementary Tables 1-2 & Supplementary Figures 1-5. [file 41598_2020_62881_MOESM1_ESM.pdf]

Oral therapy with colonization factor antigen I prevents development of type 1 diabetes in Non-obese Diabetic mice

Andrew S. Nelson[1], Department of Infectious Diseases & Immunology, University of Florida, asnelson6034@ufl.edu

Massimo Maddaloni[1], Department of Infectious Diseases & Immunology, University of Florida, maddalonim@ufl.edu

Jeffrey R. Abbott[2], Department of Comparative, Diagnostic & Population Medicine, University of Florida, abbottj@ufl.edu

Carol Hoffman[1], Department of Infectious Diseases & Immunology, University of Florida, riccardic@ufl.edu

Ali Akgul[1], Department of Infectious Diseases & Immunology, University of Florida, aliakgul@ufl.edu

Christina Ohland[3], Division of Gastroenterology, Hepatology, and Nutrition, Department of Medicine, University of Florida, Christina.Ohland@medicine.ufl.edu

Raad Z. Gharaibeh, Division of Gastroenterology, Hepatology, and Nutrition, Department of Medicine, University of Florida, raad.gharaibeh@medicine.ufl.edu

Christian Jobin[1,3], Department of Infectious Diseases & Immunology; Division of Gastroenterology, Hepatology, and Nutrition, Department of Medicine, University of Florida, Christian.Jobin@ufl.edu

Todd M. Brusko[4], Department of Pathology, Immunology, & Laboratory Medicine, University of Florida Diabetes Institute, tbrusko@ufl.edu

David W. Pascual[1]*, Department of Infectious Diseases & Immunology, University of Florida, pascuald@ufl.edu

***Correspondence:** Address inquiries and requests to D.W.P. (email: pascuald@ufl.edu)

[1]Department of Infectious Diseases and Immunology, University of Florida, Gainesville, FL, United States

[2]Department of Comparative, Diagnostic, and Population Medicine, University of Florida, Gainesville, FL, United States

[3]Division of Gastroenterology, Hepatology, and Nutrition, Department of Medicine, University of Florida, Gainesville, FL, United States

[4]Department of Pathology, Immunology, & Laboratory Medicine, University of Florida Diabetes Institute, University of Florida, Gainesville, FL, United States

Supplemental Table 1. Antibodies used for Flow Cytometry in LL-CFA/I Treg Studies.

Antibody specificity	Clone	Source	Dilution
**CD19**	EBio1D3	eBioscience	1/600
**CD25**	PC61.5	Biolegend	1/500
**CD39**	24DMS1	eBioscience	1/500
**CD4**	RM4-5	Biolegend	1/500
**CD49B**	HMa2	BD	1/400
**CD8α**	53-6.7	eBioscience	1/500
**CTLA-4**	UC10-4F10-11	BD	1/500
**Foxp3**	FJK-16s	eBioscience	1/250
**IFN-γ**	XMG1.2	BD	1/250
**IL-10**	JES5-16E3	eBioscience	1/250
**Lag-3**	C9B7W	Biolegend	1/400
**PD-1**	RMPI-30	eBioscience	1/500
**Tbet**	4B10	Biolegend	1/250
**TCR-β**	H57-597	Biolegend	1/500
**TGF-β (Lap)**	TW7-16B4	Biolegend	1/400
**TIGIT**	1G9	Biolegend	1/400
**TNF-α**	MP6-XT22	Biolegend	1/250

Summary of specificity, clone, and source of antibodies used in flow cytometry analysis.

Supplemental Table 2. Primer sequences for detection of cytokine-specific mRNA.

Gene Name	Primer	Size	Sequence (5`-3`)
**IL-6**	F	23	TAGTCCTTCCTACCCCAATTTCC
	R	21	TTGGTCCTTAGCCACTCCTTC
**TNF-α**	F	23	CCCTCACACTCAGATCATCTTCT
	R	19	GCTACGACGTGGGCTACAG
**IL-10**	F	21	GCTCTTACTGACTGGCATGAG
	R	20	CGCAGCTCTAGGAGCATGTG
**IL-33**	F	21	ACAGATATATGACTTACGGCG
	R	23	AAATGGACCCTCTCTAAAGCAAA
**GAPDH**	F	20	ACCACAGTCCATGCCATCAC
	R	19	TCCACCACCCTGTTGCTGTA
**B-actin**	F	22	ATCTACGAGGGCTATGCTCTCC
	R	21	AGCCTCGGTCAGGATCTTCAT

Supplemental Figure 1. Negative Controls for Tetramer Staining

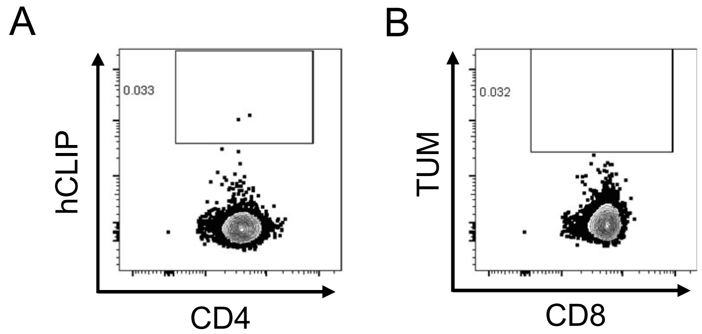

Supplemental Figure 1. Negative controls for tetramer staining. Four wk-old NOD females were orally dosed with $5x10^7$ CFUs of LL-CFA/I, LL vector, or PBS (n=5/group). Additional doses were given every 2 wks. At 11 wks of age, lymphocytes from the PaLNs were isolated and labeled with tetramers specific for (A) human CLIP or (B) TUM peptide.

Supplemental Figure 2. LL-CFA/I does not Induce Negative Regulators in NOD Mice

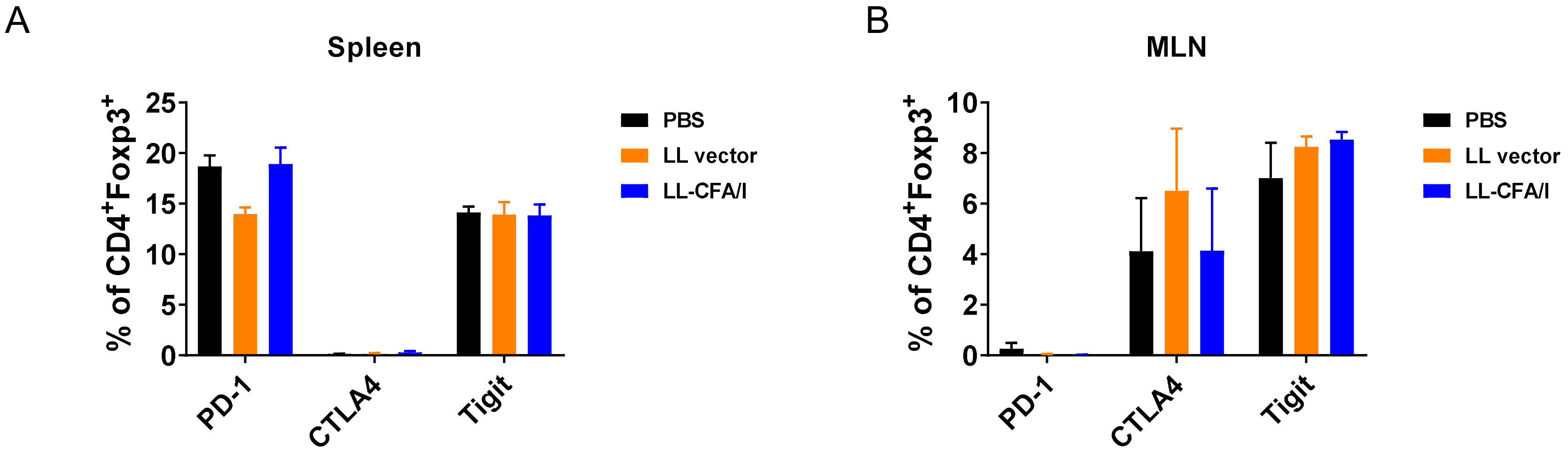

Supplemental Figure 2. LL-CFA/I does not induce negative regulators in NOD mice. Four wk-old NOD females were orally dosed with 5×10^7 CFUs of LL-CFA/I, LL vector, or PBS (n=5/group). Additional doses were given every 2 wks. At 11 wks of age, $Foxp3^+CD4^+$ Tregs from the (A) spleens and (B) MLNs were examined for expression of PD-1, CTLA-4 and Tigit.

Supplemental Figure 3. LL-CFA/I promotes regulatory environment in the gut.

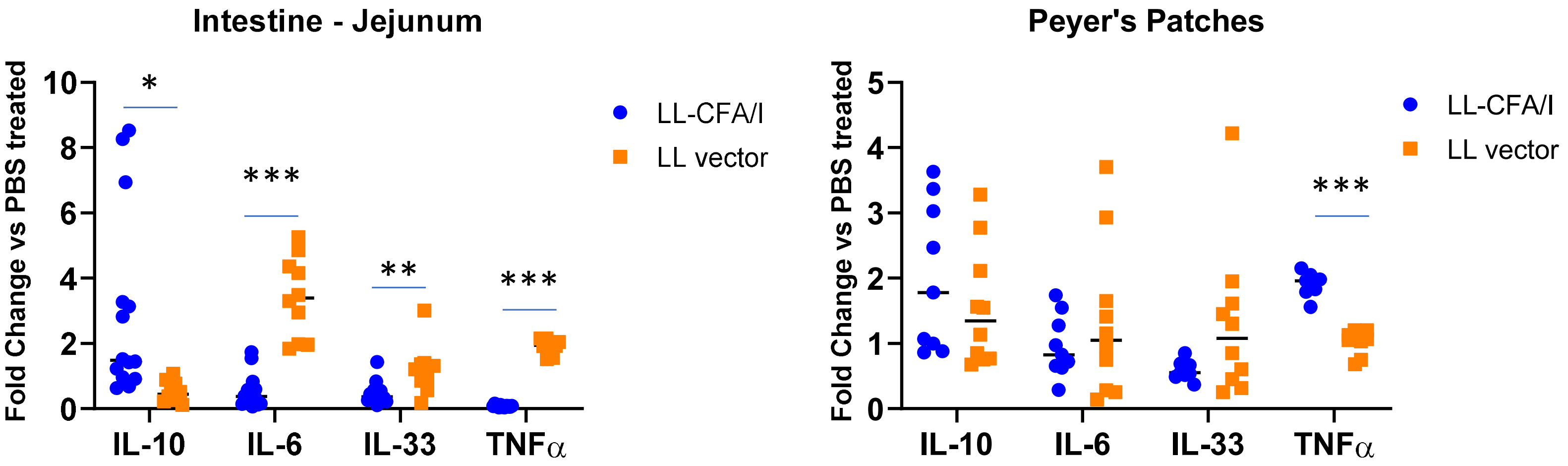

Supplemental Figure 3. LL-CFA/I promotes regulatory environment in the gut. Eight week old BALB/c mice (n=9-14/group) were orally dosed with 2×10^9 CFUs of LL-CFA/I, LL-vector or PBS. After 1.5 hours, small intestines and Peyer's Patches were collected. Expression of cytokines was analyzed as fold change against the PBS control group. **p*<0.05, ***p*<0.005, and ****p*<0.0001 for LL-CFA/I vs LL-vector.

Supplemental Figure 4. Mouse Microbiota are not Significantly Different Between Groups Before LL Treatments Begin.

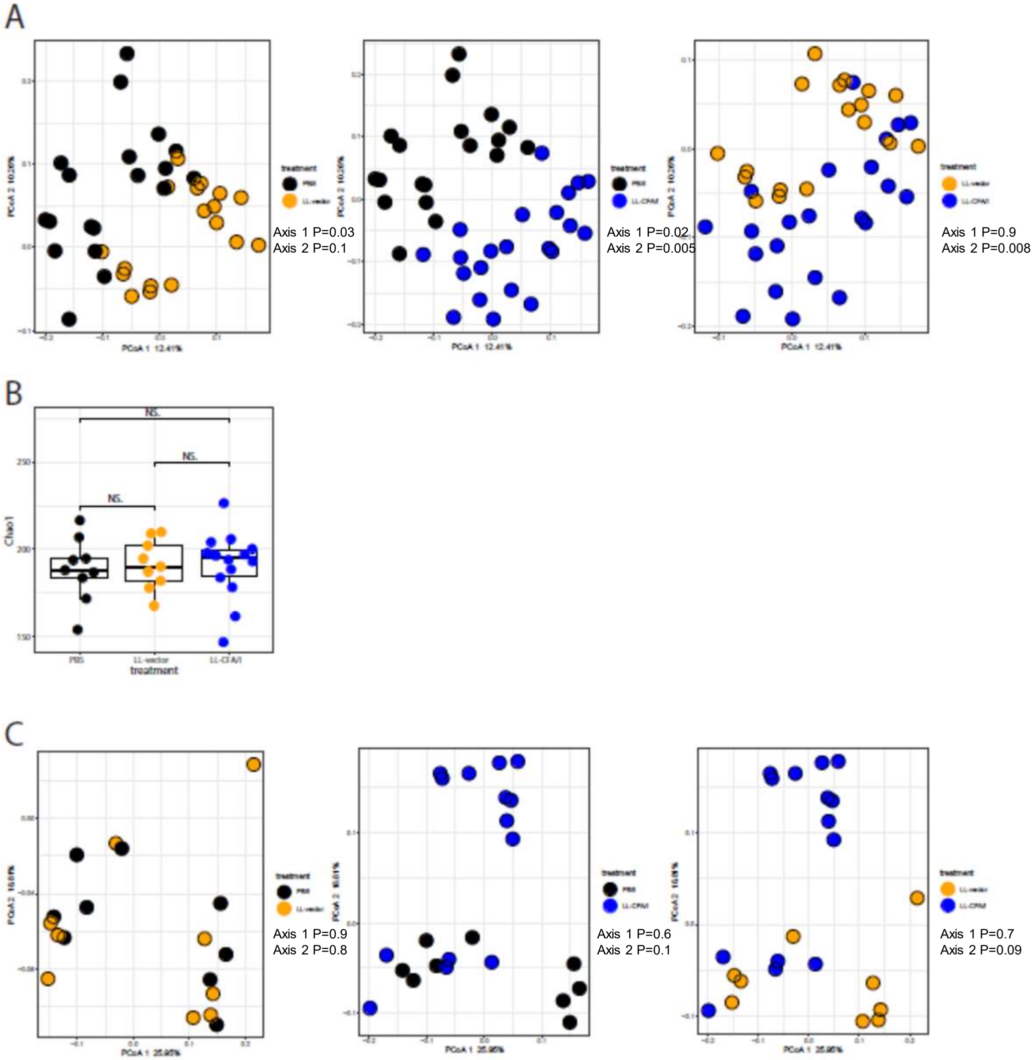

Supplemental Figure 4. Mouse microbiota are not significantly different between groups before LL treatments begin. (A) Pair-wise comparisons of beta diversity post-treatment (11 weeks old). (B) Alpha diversity of pre-treatment samples at 4 weeks old. (C) PCoA plots of beta diversity in pre-treatment samples. NS, not significant.

Supplemental Figure 5. LL-CFA/I does not Induce Splenic IFN-γ^{+}IL-10^{+} Foxp3^{+}CD4^{+} Tr1 cells at 17 weeks

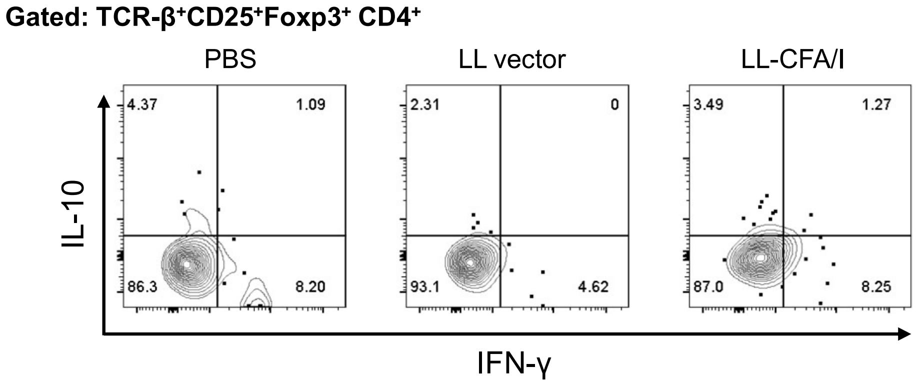

Supplemental Figure 5. LL-CFA/I does not Induce Splenic IFN-γ^{+}IL-10^{+} Foxp3^{+}CD4^{+} Tr1 cells at 17 weeks. Four wk-old NOD females were orally dosed with 5x10^{7} CFUs of LL-CFA/I, LL vector, or PBS (n=5/group). Additional doses were given every 2 wks from 6 to 16 weeks of age. (A) Splenocytes were stimulated with anti-CD3 and anti-CD28 mAbs and CD25^{+}Foxp3^{+}CD4^{+}Tregs analyzed for expression of IFN-γ and IL-10.
